# RETRACTION: Chidamide Inhibits Glioma Cells by Increasing Oxidative Stress via the miRNA-338-5p Regulation of Hedgehog Signaling

**DOI:** 10.1155/omcl/9783207

**Published:** 2025-08-01

**Authors:** Oxidative Medicine and Cellular Longevity

RETRACTION: H. Zhou, L. Han, H. Wang, J. Wei, Z. Guo, and Z. Li, “Chidamide Inhibits Glioma Cells by Increasing Oxidative Stress via the miRNA-338-5p Regulation of Hedgehog Signaling,” *Oxidative Medicine and Cellular Longevity* 2020 (2020): 7126976, https://doi.org/10.1155/2020/7126976.

The above article, published online on 11 March 2020 in Wiley Online Library (wileyonlinelibrary.com), has been retracted by agreement between the authors, the Chief Editor, Jeannette Vasquez-Vivar, and John Wiley & Sons Ltd. The retraction has been agreed due to errors in the data analysis identified by the authors, which subsequently affects the conclusions.

During further investigation by the publisher, it was also noted that in Figure 5a the CHIG panel is a duplicate of the IG panel, which has been rotated 180 degrees.

